# Shape-Depended Biological Properties of Ag_3_PO_4_ Microparticles: Evaluation of Antimicrobial Properties and Cytotoxicity in *In Vitro* Model—Safety Assessment of Potential Clinical Usage

**DOI:** 10.1155/2019/6740325

**Published:** 2019-11-20

**Authors:** Karol P. Steckiewicz, Julia Zwara, Maciej Jaskiewicz, Szymon Kowalski, Wojciech Kamysz, Adriana Zaleska-Medynska, Iwona Inkielewicz-Stepniak

**Affiliations:** ^1^Department of Medical Chemistry, Medical University of Gdansk, Faculty of Medicine, Gdansk, Poland; ^2^Department of Histology, Medical University of Gdansk, Faculty of Medicine, Gdansk, Poland; ^3^Department of Environmental Technology, Faculty of Chemistry, University of Gdansk, Gdansk, Poland; ^4^Department of Inorganic Chemistry, Faculty of Pharmacy, Medical University of Gdansk, Gdansk, Poland

## Abstract

Implant-related infections are an emerging clinical and economic problem. Therefore, we decided to assess potential clinical usefulness and safety of silver orthophosphate microparticles (SOMPs) regarding their shape. We synthesized and then assessed antimicrobial properties and potential cytotoxicity of six shapes of SOMPs (tetrapod, cubes, spheres, tetrahedrons, branched, and rhombic dodecahedron). We found that SOMPs had a high antimicrobial effect; they were more efficient against fungi than bacteria. SOMPs exerted an antimicrobial effect in concentrations not toxic to mammalian cells: human fetal osteoblast (hFOB1.19), osteosarcoma (Saos-2), mouse preosteoblasts (MC3T3-E1), skin fibroblast (HDF), and mouse myoblast (C2C12). At higher concentration SOMPs, induced shape- and concentration-dependent cytotoxicity (according to MTT and BrdU assays). Tetrapod SOMPs had the smallest effect, whereas cubical SOMPs, the highest on cell viability. hFOB1.19 were the most resistant cells and C2C12, the most susceptible ones. We have proven that the induction of oxidative stress and inflammation is involved in the cytotoxic mechanism of SOMPs. After treatment with microparticles, we observed changes in levels of reactive oxygen species, first-line defense antioxidants-superoxide dismutase (SOD1, SOD3), and glutathione peroxidase (GPX4), metalloproteinase (MMP1, MMP3), and NF-*κ*B protein. Neither cell cycle distribution nor ultrastructure was altered as determined by flow cytometry and transmission electron microscopy, respectively. In conclusion, silver orthophosphate may be a safe and effective antimicrobial agent on the implant surface. Spherical-shaped SOMPs are the most promising for biomedical application.

## 1. Introduction

Nowadays, due to the development of medicine, life span and quality increased. Unfortunately, to achieve that goal, the patient sometimes needs to undergo surgery with implantation of a foreign body (e.g., valve or joint replacement and bone fracture treatment). These procedures are not complication-free and among many others, the infections may appear. Implant-related infections are a severe clinical and epidemiological problem, which can occur up to 3-5% of orthopedic patients and can affect even up to 40% of patients with cardiovascular implants (regardless of prophylaxis) [1, 2]. Among several etiological factors of those infections, *Staphylococcus aureus*, especially methicillin-resistant strains (MRSA) and fungi like *Candida albicans* and *Aspergillus niger* are the most common [2, 3]. As a matter of fact, in foreign bodies, there is no microcirculation, which is crucial for host defense and drug delivery [3]. Furthermore, medical devices (implants, bone nails, vascular grafts, artificial valves, etc.) can be easily colonized by pathogens and lead to biofilm formation. Biofilm can be described as a complex structure consisting of high-concentration tightly attached bacterial cells and extracellular matrix; therefore, antibodies or drugs poorly penetrate it [4]. Furthermore, biofilm can produce substances which will deactivate antimicrobial agents, which makes treatment less likely to succeed [2, 4]. This should be noted that planktonic forms of bacteria can be 100-1000 times more susceptible to antimicrobial substances compared to those in biofilm form [5]. Implant-related infections are treated by either antibiotic therapy, surgery, or both. Unfortunately, this medical condition is fatal even in 30% of patients with prosthetic valve endocarditis [2, 5]. Moreover, only in the US treatment of all implant-related infection costs around 3.3 billion USD annually (1.86 billion USD, orthopedic implants-related infection alone) [2]. Thus, it is also a major economic issue. However, apart from emphasizing the importance of aseptic surgery techniques, any new solution to that matter has not been recently proposed [6].

Thus, novel approaches are being searched. Recently Zhang et al. reported that nanohydroxyapatite/polyurethane/silver composite may be successfully used to treat osteomyelitis in rabbits [7]. Also Jinag et al. suggested nanohydroxyapatite/polyurethane/silver phosphate composite as an antibacterial agent [8]. In another study, calcium phosphate/silver biomaterial has been proposed as antibacterial implant coating [9]. Similarly, calcium phosphate/silver phosphate particles may be used in dentistry as an antibacterial and remineralising factor [10].

Silver orthophosphate microparticles (SOMPs) may be an interesting solution to implant-related infections but their usefulness is yet to be examined. Antimicrobial properties of silver are well known, and the presence of phosphorus in the compound may increase biocompatibility [11–13]. Firstly discovered by Yi et al., SOMPs currently are studied as photocatalysts [14]. Their photocatalytic activity under visible light is used to remove pollution from the natural environment [15]. In this study, we aim to determine whether antimicrobial properties and cytotoxicity of silver phosphate microparticles are shape-dependent. It has been proven that chemical properties of SOMPs are shape-dependent. Their photocatalytic activity is reliant on surface morphology and properties [16]. Therefore, we hypothesize that the difference in surface properties of shapes of SOPMs will have an impact on their characteristics in *in vitro* systems. We synthesized six shapes of SOMPs (tetrapod, cubes, spheres, branches, tetrahedrons, and rhombic dodecahedrons) and examined them in *in vitro* model. Potential clinical usefulness and safety of application were taken into concern. According to our best knowledge, it is the first study in which either silver phosphate nanoparticles (SONPs) or SOMPs were studied in mammalian cell lines.

## 2. Materials and Methods

### 2.1. Materials and Instruments

The silver nitrate (98%, Sigma-Aldrich) was used as a precursor for the synthesis of Ag_3_PO_4_ powder. PVP (Mw = 300,000), sodium dihydrogen phosphate dihydrate (NaH_2_PO_4_·2H_2_O, 99%), sodium phosphate decahydrate (Na_3_PO_4_·10H_2_O, 99%), N,N-dimethylformamide (DMF), hexamethylenetetramine (HMT), and urea (CO(NH_2_)_2_) were purchased from Sigma-Aldrich. Phosphoric acid (H_3_PO_4_, 85%), ammonia (NH_3_·H_2_O, 10%), and ethanol (CH_3_CH_2_OH, 96%) were purchased from POCH S.A., Poland. All chemicals were used without further purification. The morphology of Ag_3_PO_4_ semiconductors was measured by scanning electron microscope (SEM, JEOL JSM-7610F) working in high vacuum mode. DRS UV–Vis spectra of the synthesized samples were recorded in the scan range 300–700 nm using UV–Vis spectrophotometer (Evolution 220, Thermo Scientific) and BaSO_4_ as the reference.

### 2.2. Synthesis of Different Shapes of SOMPs

The spherical SOMPs (s-SOMPs) were obtained by a chemical precipitation method [17]. In the first step, 7.9416 g of polyvinylpyrrolidone (PVP) was dissolved in 200 mL of deionized water. Then, 0.4246 g of AgNO_3_ was dissolved in 100 mL of deionized water and added to the PVP solution. Aqueous Na_2_HPO_4_ solution (0.5678 g in 200 mL) was added dropwise and stirred until the solution turned yellow. The resulting yellow precipitate was separated by centrifugation, washed 3 times with deionized water and ethanol, and then dried in a vacuum oven at 60°C until the liquid completely evaporated. The cubic SOMPs (c-SOMPs) were obtained by the ion exchange method [15]. 0.5096 g AgNO_3_ was dissolved in 90 mL of deionized water under stirring. A solution of aqueous ammonia was added to the solution thus prepared to obtain a brown solid completely dissolved in the solution. The next step was to add 0.1639 g of Na_3_PO_4_ dissolved in 30 mL of deionized water. After stirring for 5 minutes, the precipitate was collected, washed several times with deionized water, and dried in a desiccator. The tetrahedral SOMPs (th-SOMPs) were obtained by the soft chemical method [18]. First, 10 mL of N,N-dimethylformamide (DMF) with 10 mL of deionized water were mixed. 0.5096 g AgNO_3_ was added to the above transparent solution and then 1 mL H_3_PO_4_ was added dropwise. The resulting mixture was sonicated for 2 h. Ag_3_PO_4_ microcrystals were collected, washed several times with distilled water and ethanol to remove DMF residues, and dried in a vacuum oven overnight at 80°C. Rhombic dodecahedral SOMPs (rd-SOPMs) were obtained by the hydrothermal method [19]. In the first step, 1.34 g of AgNO_3_ was dissolved in 10 mL of deionized water. Then, 0.92 g (0.0006 mol) NaH_2_PO_4_·2H_2_O was dissolved in 6 mL of deionized water and added dropwise to the AgNO_3_ solution. The solution was allowed to stir for 5 minutes. After this time, an aqueous solution of ammonia was added until the pH was adjusted to 7. The resulting mixture was transferred into a Teflon-lined stainless steel autoclave and treated at 160°C for 6 h. After cooling to room temperature, the yellow precipitate was separated by centrifugation, washed three times with deionized water and methanol, and dried overnight at 60°C. Branched SOMPs (b-SOMPs) were obtained by a chemical precipitation method [20]. 0.318 g of AgNO_3_ was dissolved in 40 mL of deionized water, and then 41 *μ*L of 85 wt.% H_3_PO_4_ was added dropwise. In the next step, 0.197 g of hexamethylenetetramine was added to the solution and mixed for 5 minutes to change color to yellow. The resulting precipitate was collected, washed with deionized water3 times, and dried in a vacuum oven at 60°C. Tetrapod SOMPs (t-SOMPs) were obtained by the hydrothermal method [21]. In the first step, 3 mmol of 85 wt.% H_3_PO_4_ and 2.5 mmol AgNO_3_ were dissolved in 80 mL of deionized water. 37.5 mmol of urea was added to the above solution and mixed for 5 minutes until complete dissolution. Immediately afterward, the resulting mixture was transferred into a Teflon-lined stainless steel autoclave and kept at 80°C for 24 h. The yellow powder was separated by centrifugation, washed 3 times with deionized water and ethanol, and then dried overnight at 60°C.

### 2.3. Reference Strains of Microorganisms

Reference strains of staphylococci, namely *Staphylococcus aureus* ATCC 25923 and MRSA ATCC 33591, before the tests were cultivated in Mueller–Hinton Broth (BioMaxima, Lublin, Poland) for 24 hours with shaking. For fungi, *Candida albicans* ATCC 10231 and *Aspergillus niger* ATCC 16404, the cultivation was held in the RPMI 1640 medium (Sigma-Aldrich, Steinheim, Germany) for 24 hours and 5 days, respectively.

### 2.4. Determination of Antimicrobial Activity

The minimal inhibitory concentrations (MICs) for bacteria and fungi were determined by the broth microdilution method according to the Clinical and Laboratory Standards Institute (CLSI) recommendation [22, 23]. For this purpose, the initial inoculums of bacteria (5 × 10^5^ CFU/mL) in Mueller–Hinton Broth were exposed to the ranging concentrations of the test compounds (1–512 *μ*g/mL) and incubated for 18 h at 37°C. For fungi, the initial inoculums of 2 × 10^3^ CFU/mL in RPMI 1640 were exposed to the ranging concentrations of the test compounds (1–256 *μ*g/mL) and incubated at 37°C for 24 h and 48 h, respectively. The experiments were conducted on 96-well microtiter plates, with the final volume of 100 *μ*L. Cell densities were adjusted spectrophotometrically (Multiskan™ GO Microplate Spectrophotometer, Thermo Scientific) at the wavelengths of 600 nm for bacteria and 530 nm for fungi. The MIC was taken as the lowest drug concentration at which a noticeable growth of microorganisms was inhibited.

Minimum biofilm eradication concentrations (MBECs) were determined as previously described [11, 24, 25]. For this purpose, 96-well polystyrene flat-bottom plates and a resazurin (7-hydroxy-3H-phenoxazin-3-one 10-oxide) as a cell viability reagent were used. In this assay, a specific feature of resazurin is utilized, which upon the contact with living cells is metabolized and reduced from the basic blue form to pink resorufin. Briefly, the preprepared cultures of microorganisms were diluted to obtain the final density of 5 × 10^5^ CFU/mL in Mueller–Hinton Broth for bacteria and 2 × 10^5^ CFU/mL in RPMI-1640 for fungi per well (100 *μ*L). After 24 h of incubation at 37°C, the wells of the plates were rinsed three times with phosphate buffer saline (PBS) to remove nonadherent cells. Subsequently, 100 *μ*L of tested compounds in a concentration range (diluted in appropriate media) was added to each well. After 24 h of incubation at 37°C, 20 *μ*L of the resazurin (4 mg/mL) was added. The MBEC was read after 1 h. MBECs were determined as the lowest concentration at which the reduction of resazurin was lower or equal (10% ± 0.5%) as compared to positive (100%) and negative (0%) controls. All experiments were performed in triplicate using Multiskan™ GO Microplate Spectrophotometer.

### 2.5. Cell Culture

hFOB 1.19 (human fetal osteoblast), MC3T3-E1 (mouse preosteoblast), SaoS-2 (human osteosarcoma), C2C12 (mouse myoblast), and HDF (human dermal fibroblasts) cells were used in the study. hFOB 1.19 (ATCC CRL-11372) were cultured in a 1 : 1 mixture of Ham's F12 Medium Dulbecco's Modified Eagle's Medium supplemented with 2.5 mM L-glutamine, 10% fetal bovine serum (FBS), and 1% of penicillin/streptomycin (P/S). MC3T3-E1 subclone 4 (ATCC CRL-2593) were cultured in the Alpha Minimum Essential Medium with ribonucleosides, deoxyribonucleosides, 2 mM L-glutamine, and 1 mM sodium pyruvate, 10% of FBS, and 1% of P/S, but without ascorbic acid. Saos-2 (ATCC HTB-85) were cultured in McCoy's 5a Medium Modified supplemented with 15% of FBS and 1% of P/S. C2C12 (ECACC no. 91031101) were cultured in high-glucose Dulbecco's Modified Eagle's Medium (DMEM) supplemented with 10% of FBS and 1% of P/S. HDF cells were cultured in high-glucose DMEM supplemented with 10% of FBS and 1% of P/S. All cells were cultured under sterile condition. Cells were kept at 37°C in a humidified atmosphere of 5% CO_2_. Cells were maintained in 75 cm^2^ tissue culture flask. The medium was replaced every 48 h. When confluent, cells were detached with a trypsin-EDTA solution and subcultured into a newer flask.

### 2.6. Treatments

hFOB1.19, MC3T3-E1, Saos-2, C2C12, and HDF cells were treated with different shapes of SOMPs for 24 h. Concentrations used in experiments were determined by preliminary studies. Each time, just before, experiment SOMPs were diluted in FBS-free media and shaken well to ensure equal dispersion of SOMPs in solution. Control samples were treated with SOMPs-free and FBS-free culture media. During the incubation process, the medium was not changed.

### 2.7. MTT Viability Assay

hFOB1.19, MC3T3-E1, Saos-2, C2C12, and HDF cells were used in the assay. Cells were seeded in 96-well plates. After 24 h of incubation, media were changed and cells were treated with microparticles in the concentration range of 0.01-10 *μ*g/mL as described in Treatments. After 24 h, media were supplemented with water-soluble tetrazolium salt (final concentration 0.5 mg/mL) and incubated for 2 h. Next, media were removed and crystals were dissolved in DMSO. After 15 min, cell viability was assessed by measuring absorbance at 540 nm (reference 630 nm) using a microplate reader. Viability was determined as a percentage of control (viability of control cells was set as 100%). Absorbance values were corrected with blank microparticles.

### 2.8. BrdU Proliferation Assay

BrdU proliferation Elisa kit (Roche) was used to measure cell proliferation. hFOB1.19, MC3T3-E1, SaoS-2, C2C12, and HDF-1 cells were used in the assay. Cells were seeded in 96-well dish and treated with microparticles, in a concentration range 0.01-10 *μ*g/mL as described in Treatments. Next, the antiproliferative activity of microparticles was measured by BrdU incorporation according to the manufacturer protocol. Data are shown as a percentage of control (proliferation rate of control cells was set as 100%). Absorbance values were corrected with blank microparticles.

For ROS detection, flow cytometry, and Western blotting, we decide to use two cell lines. hFOB1.19 and C2C12 cells had been chosen due to their different molecular characteristic and response to SOMPs in the preliminary study. Based on the antimicrobial assay and preliminary cytotoxicity studies for those assays, we decided to use three shapes (c-SOMPs, s-SOMPs, and b-SOPMs).

### 2.9. Detection of Reactive Oxygen Species

hFOB1.19 and C2C12 were seeded into 6-well plates; the next day, the medium was replaced and cells were treated with selected shapes as described in Treatments. Cells were treated with microparticles in 1, 3, and 5 *μ*g/mL concentrations. After the incubation, media were discarded and replaced with a new solution supplemented with 10 *μ*M 2,7-dichlorofluorescein diacetate (DCF-DA). After 30 min, fluorescence of oxidized DCF was measured by flow cytometry (excitation wavelength: 480 nm; an emission wavelength: 525 nm). Data were expressed as a percentage of untreated cells (which was set as 100%).

### 2.10. Cell Cycle Analysis

hFOB1.19 and C2C12 were seeded into 6-well plates and treated with SOMPs in 3 and 5 *μ*g/mL concentrations for 24 h as described in Treatments. After incubation, cells were washed, harvested, and fixed (70% ethanol, 4°C). Next, cells were centrifuged and suspended in PBS with RNAse A (50 *μ*g/mL) and propidium iodide (50 *μ*g/mL). After 30 min, samples were analyzed by flow cytometry (BD FACSCalibur™, CellQuest Pro software). FSC/SSC and FL2-A/FL2-W plots were gated to avoid doublets and debris. The number of cells in each cell cycle phase was determined by software usage (sample size of at least 15,000 cells).

### 2.11. Western Blotting

Western blot analysis was performed to determine the impact of SOMPs on SOD1 (superoxide dismutase [Cu-Zn]), SOD2 (mitochondrial superoxide dismutase), SOD3 (extracellular superoxide dismutase [Cu-Zn]), GPX4 (glutathione peroxidase 4), NF-*κ*B (nuclear factor kappa-light-chance-enhancer of activated B cells), MMP-1 (matrix metalloproteinase 1), MMP-3 (matrix metalloproteinase 3), and p16-ARC (human p16 actin-related complex) expressions. The method was previously established and described [26]. Briefly, hFOB1.19 and C2C12 cells were seeded into 100 mm Petri dishes. When the confluent medium was changed and cells were treated with c-SOMPs, s-SOMPs, or b-SOPMs in 3 and 5 *μ*g/mL concentrations as described in Treatments. After 24 h, the medium was removed and cells were washed, detached, and lysed. Next, protein levels were measured by the Bradford method [27], samples prepared, and electrophoresis performed. After electrophoresis, proteins were transferred onto nitrocellulose membranes (Protran®, Schleicher and Schuell BioScience) and detected using antibodies. *β*-Actin was used as a loading control. The immunoactive proteins were detected using an enhanced chemiluminescence Western blotting detection kit (Amersham Biosciences, Piscataway, NJ, USA). Protein levels were quantified using densitometry software (ImageLab, Bio-Rad).

### 2.12. Transmission Electron Microscopy

Transmission electron microscopy (TEM) was used to determine SOMP uptake and ultrastructure changes in the cells. C2C12 cells were used for TEM analysis. As previously described [26], cells were plated into 100 mm Petri dishes. After 24 h, cells were treated with c-SOMPs, s-SOMPs, or b-SOPMs in a concentration of 3 *μ*g/mL as described in Treatments. Next, cells were fixed (2.5% glutaraldehyde in 0.1 mM sodium-cacodylate buffer), detached, and centered. The cell pellets were postfixed (2% osmium tetroxide) and dehydrated (graded series of ethanol). After infiltration (propylene dioxide: epon mixture, pure epon), pelleted cells were embedded to polymerize. Finally, the ultrathin sections (Reichert OmU3 Ultramicrotome, Austria) were contrasted (uranyl acetate, lead citrate) prior to examination in transmission electron microscope at 100 kV (JEM 1200EX II, Jeol, Japan).

### 2.13. Statistical Analysis

Data are shown as the mean ± standard error of 4 independent experiments. Statistical analysis was determined by one-way analysis of variance (ANOVA) and Tukey's post hoc test. The IC_50_ was calculated by analyzing a nonlinear regression log(inhibitor) vs. normalized response. Statistical analysis was made with GraphPad5 software.

## 3. Results

### 3.1. Morphology of Silver Phosphate Microparticles

**Figure 1:**
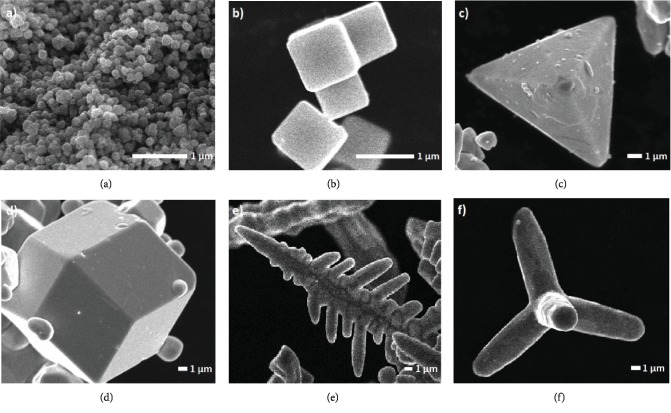
SEM images of Ag_3_PO_4_ at different shapes: (a) s-SOMPs, (b) c-SOMPs, (c) th-SOMPs, (d) rd-SOMPs, (e) b-SOMPs, and (f) t-SOMPs particles.

The SEM images of the samples obtained are shown in Figure 1. s-SOMPs (Figure 1(a)) are characterized by an irregular shape with a particle diameter of approximately 500 nm. s-SOMPs also show a tendency to rapid nucleation and particle growth, which leads to their agglomeration. In this case, the geometrical shape and size of the particles are responsible for PVP, which is added at the stage of synthesis [17]. c-SOMPs are shown in Figure 1(b). The structure is characterized by a smooth surface ending with sharp edges with an average length of 1-1.5 *μ*m. In this case, the addition of ammonia during the synthesis led to the formation of c-SOMPs [28]. The characteristic morphology of th-SOMPs are demonstrated in Figure 1(c). The SEM image shows the high efficiency of forming structures with sharp corners, edges, and smooth surfaces. Furthermore, a polyhedron with four triangular walls has side lengths from 4 to 0.5 *μ*m. Dong et al. also synthesized Ag_3_PO_4_ particles; however, their length was from 0.5 to 1 *μ*m, and the lateral edges and vertices were rounded [29]. Wan et al. received crystals with an average size of 740 nm [30].  Figure 1(d) shows the rd-SOMPs consisting of 12 walls, which are congruent rhombuses. The obtained structure has also a smooth surface with a diameter of 5-17 *μ*m. Dong et al. also synthesized rhombic dodecahedral crystals with a diameter of 200-600 nm, while Bi et al. with a size between 4 and 7 *μ*m [31, 32]. Typical b-SOMPs obtained under static conditions are presented in Figure 1(e). The resulting multiarmed dendrites with developed subbranches are characterized by a shoulder length of approximately 25 *μ*m. Wang et al. explained that it is impossible to obtain Ag_3_PO_4_ without addition of HMT during the synthesis because silver orthophosphate is soluble at low pH values [33]. Dong et al. obtained branched structures using a reaction solvent during the synthesis consisting of H_2_O and DMF. The length of the branches obtained was between 5 and 10 *μ*m [18]. The SEM image in Figure 1(f) shows the morphology of the t-SOMPs. t-SOMPs obtained in the presence of urea have four arms in the form of cylindrical microrods with an average diameter of 2.5 *μ*m and a length of 11-30 *μ*m. Dong et al. received silver orthophosphate in the form of a dendritic long tetrapod with a shoulder length of about 20-30 *μ*m. t-SOMPs with longer dendritic arms arose when glacial acetic acid was added to the system, acting as shape-controlling agents [29]. Based on the obtained morphology, it can be concluded that obtaining different shapes of Ag_3_PO_4_ depends on the adjustment of external experimental conditions (mixing, ultrasonic treatment), as well as through pH control or the addition of appropriate structure-controlling agents (PVP, ammonia, and HMT). The crystal structure of different Ag_3_PO_4_ shapes was characterized by pXRD in a previous work prepared by Zwara et al. [16]. The obtained results indicated the success of the experiment and obtaining Ag_3_PO_4_ crystallites. Moreover, it confirms the high purity of the samples. Additionally, pXRD reflections are sharp which suggest high crystallinity of the material.

### 3.2. Absorption Properties

**Figure 2:**
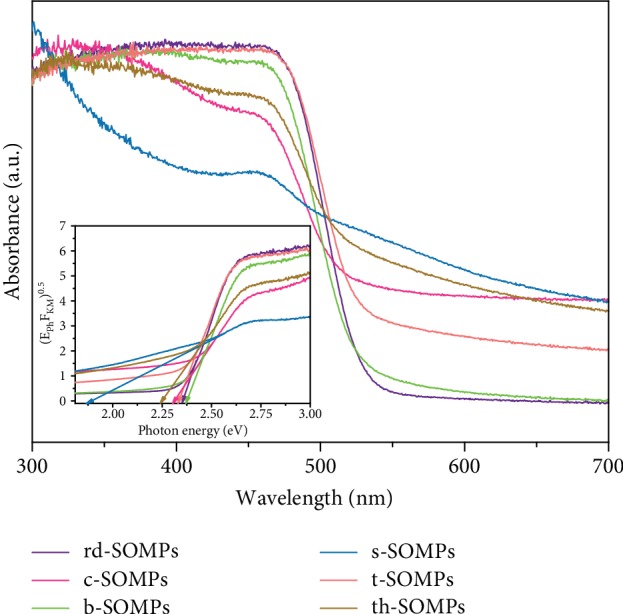
UV–Vis/DRS spectrum of Ag_3_PO_4_ photocatalysts in different shapes. Determination of the bandgap is shown in inset.

Figure 2 shows the UV–Vis/DRS absorption spectra and the Kubelka–Munk function transformation plot vs. photon energy for all as-prepared SOMPs. Analysis by UV–Vis/DRS spectroscopy has shown that SOMPs absorb irradiation in the range of around 510-590 nm. s-SOMPs and th-SOMPs absorb visible light at a wavelength less than 590 nm, while in the form of c-SOMPs at 575 nm. The spectra presented by Dong et al. show that Ag_3_PO_4_ with the structure of irregular spheres and tetrahedrons absorbs visible light with the same wavelength at 525 nm. In contrast, absorption for ankles was estimated by Bi et al. and had an edge at 520 nm [31]. t-SOMPs, rd-SOMPs, and b-SOMPs have an absorption edge at 550, 540, and 535 nm, respectively. Dong et al. also estimated the absorption edge for tetrapod and branched form at 525 nm, while absorption at wavelengths shorter than 550 nm was determined by Bi et al [31, 34]. Bandgaps of the obtained Ag_3_PO_4_ shapes are shown in Figure 2 (inset). The lowest value of the energy gap was observed for the spheres and the highest for the branched structure and was calculated to be 1.86 eV and 2.37 eV, respectively. Tetrahedrons, cubes, tetrapods, and rhombic dodecahedrals were characterized by energy bands of 2.24 eV, 2.31 eV, 2.33 eV, and 2.35 eV. The difference in the obtained values indicates the multifaceted morphology on nanoparticles. In addition, the different shapes of absorption bands, in particular Ag_3_PO_4_ spheres, may result from the content and distribution on the surface of reduced Ag metallic particles.

### 3.3. Antimicrobial Activity

All of the examined SOMPs shapes exhibited antimicrobial activity against tested staphylococci and fungi (Tables 1 and 2). Among them, the most active were c-SOMPs with the lowest minimal inhibitory concentrations of 8 *μ*g/mL against reference *S*. *aureus* ATCC 25923 and MRSA ATCC 33591 and 4 *μ*g/mL and 1 *μ*g/mL against *C*. *albicans* and *A*. *niger*, respectively. Interestingly, the antibiofilm activity of c-SOMPs was 1- to 2-fold dilution lower than in the case of MICs. Moreover, the same relation was found for other SOMPs with an exception of *S*. *aureus* ATCC 25923 strain for which MBECs of th-SOMPs, b-SOMPs, and r-SOMPs were 8 times higher than MICs. In Supplementary [Supplementary-material supplementary-material-1], we provide MIC values for clinically used antimicrobial agents as reference.

### 3.4. Cytotoxicity of SOMPs

**Figure 3:**
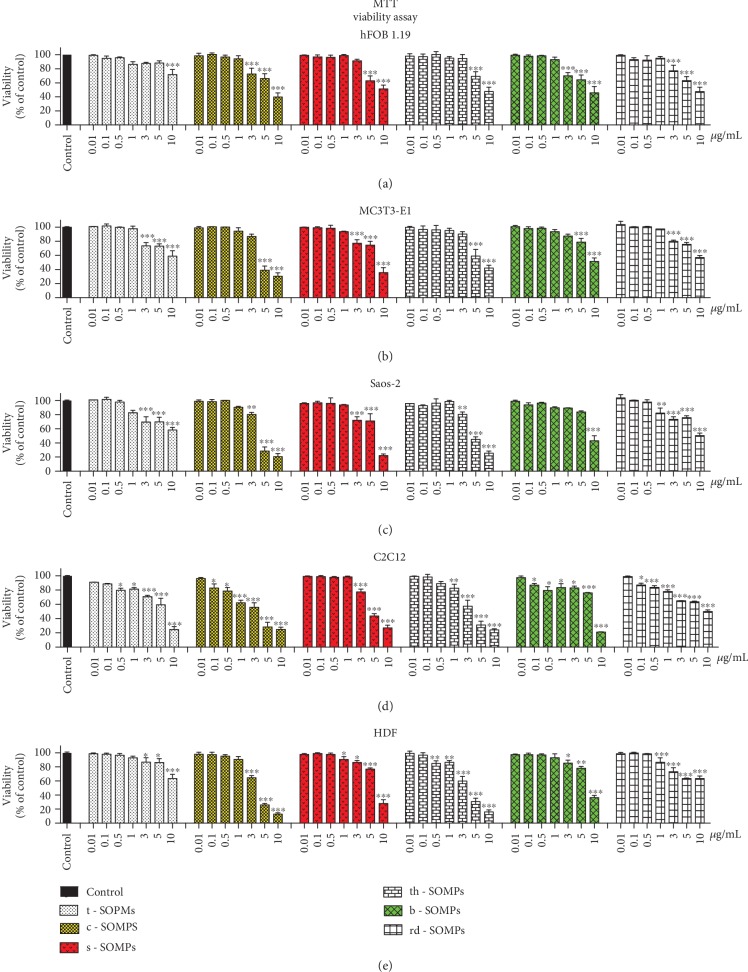
Impact of SOMPs on cell viability. Viability, measured by MTT assay of (a) hFOB1.19 cells, (b) MC3T3-E1, (c) Saos-2, (d) C2C12, and (e) HDF cells exposed to different shapes of SOMPs after 24 h. Color bars indicate shapes of SOMPs selected for further analysis. Data are presented as mean ± SD. ^∗^*p* < 0.05, ^∗∗^*p* < 0.01, ^∗∗∗^*p* < 0.001.

Figure 3 illustrates changes in the viability of the cells measured by MTT assay after treatment with different shapes of SOMPs. In Table 3, we presented IC_50_ values for SOMPs. All tested shapes decreased the viability of the cells in a concentration-dependent manner. It is clear that shape is an important modulator of SOMPs cytotoxicity. c-SOMPs were the most cytotoxic shape. In the highest tested concentration (10 *μ*g/mL), they decreased the viability of hFOB1.19 cells to around 40%, MC3T3-E1 cells to around 30%, Saos-2 and C2C12 cells to around 20%, and HDF cells to around 10%. t-SOMPs had the smallest effect on cells viability. In the highest tested concentration (10 *μ*g/mL), they decreased the viability of hFOB1.19 cells to around 75%, MC3T3-E1, and HDF cells to around 60%, Saos-2 cells to around 55%, and C2C12 cells to around 25%. It can be deducted that hFOB1.19 cells were the most resistant and C2C12 cells were the most susceptible to tested SOMPs. Importantly, SOMPs can be selectively cytotoxic only to bacteria and fungi and not harmful to mammalian cell lines. For example, c-SOMPs in MIC and MBEC concentration for *Aspergillus niger* are not cytotoxic to all cell lines apart from C2C12 cells. Moreover, there is no doubt that bacteria and fungi are more susceptible to SOMPs than mammalian cells.

### 3.5. Impact of SOMPs on Cell Proliferation

**Figure 4:**
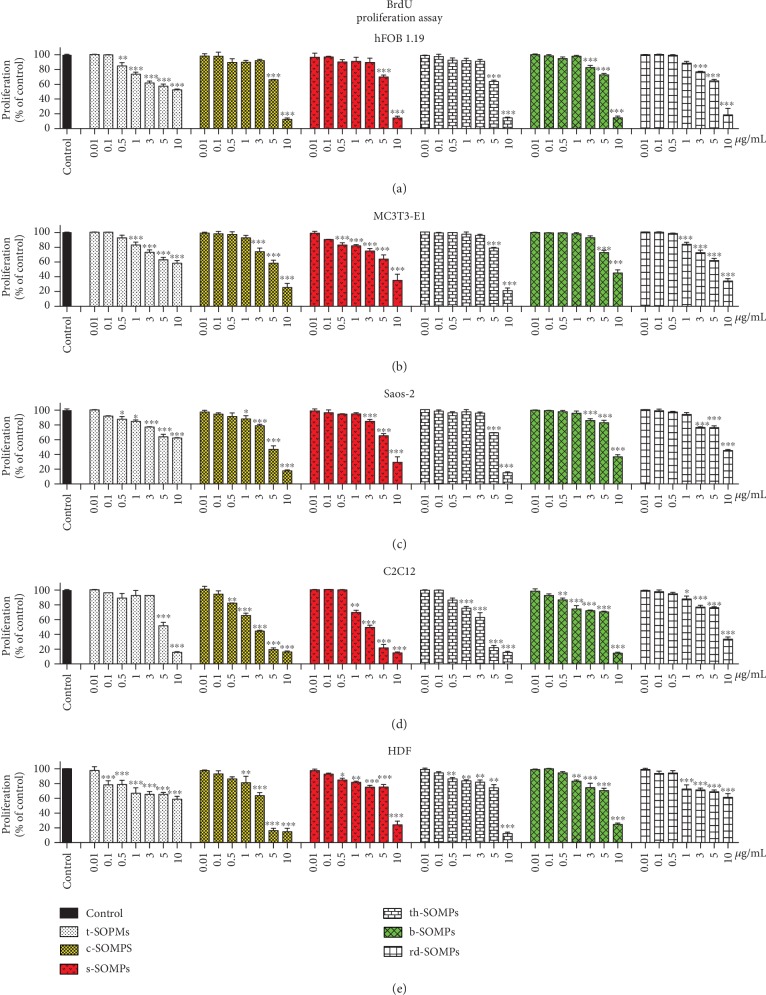
Impact of SOMPs on cell proliferation. Proliferation, measured by BrdU assay of (a) hFOB1.19 cells, (b) MC3T3-E1, (c) Saos-2, (d) C2C12, and (e) HDF cells exposed to different shapes of SOMPs after 24 h. Color bars indicate shapes of SOMPs selected for further analysis. Data are presented as mean ± SD. ^∗^*p* < 0.05, ^∗∗^*p* < 0.01, ^∗∗∗^*p* < 0.001.

Figure 4 illustrates changes in the proliferation of the cells measured by BrdU assay after treatment with different shapes of SOMPs. In Table 4, we presented IC_50_ values for the test. All SOMPs influenced cell proliferation in a concentration-dependent manner. Similar to MTT assay, t-SOMPs had the smallest effect, whereas c-SOMPs, the highest on BrdU assay results. Also, hFOB1.19 were the most resistant cells and C2C12 the most susceptible ones. Generally, SOMPs statistically significantly decreased cell proliferation (BrdU assay) in lower concentration than needed to reduce cell viability (MTT assay).

Based on antimicrobial and cytotoxicity screening, we decided to further examine three shapes of SOMPs (c-SOMPs, s-SOMPs, and b-SOPMs). They are highlighted by color bars on plots throughout Figures 3 and 4.

### 3.6. Impact of SOMPs on ROS and Oxidative Stress-Related Proteins Levels

**Figure 5:**
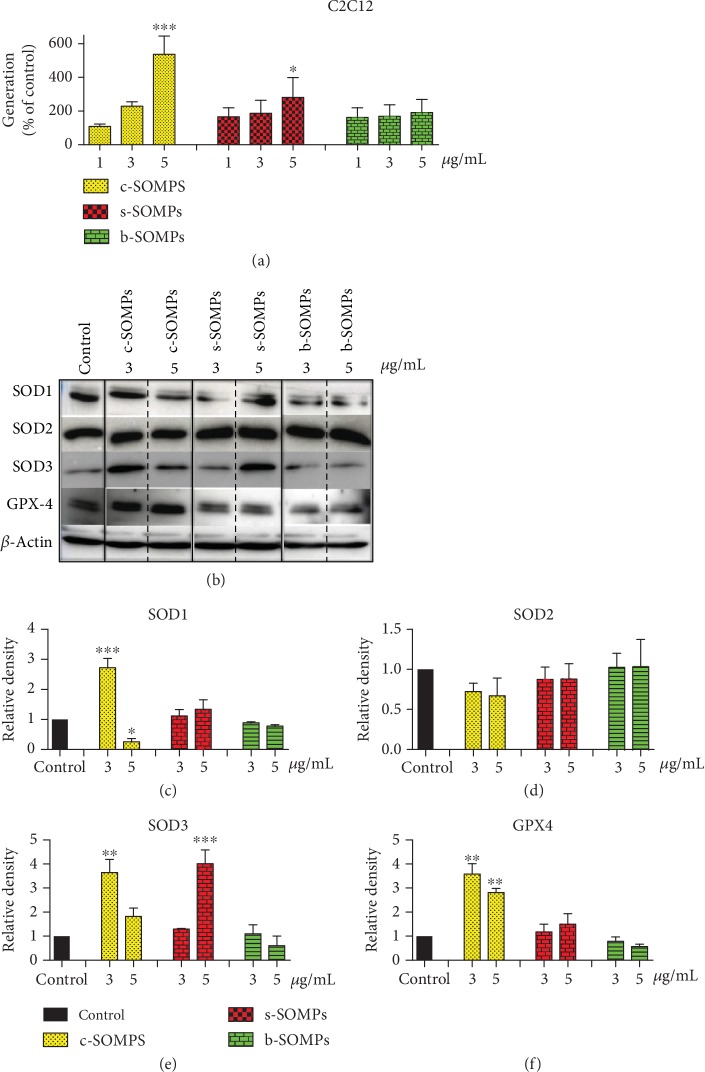
Impact of SOMPs on oxidative stress in C2C12 cells. (a) ROS levels, (b) representative Western blots, (c) quantification of SOD1 levels, (d) quantification of SOD2 levels, (e) quantification of SOD3 levels, and (f) quantification of GPX4 levels in C2C12 cells after incubation with c-SOMPs, s-SOMPs, or b-SOPMs for 24 h. Data are presented as mean ± SD. ^∗^*p* < 0.05, ^∗∗^*p* < 0.01, ^∗∗∗^*p* < 0.001.

**Figure 6:**
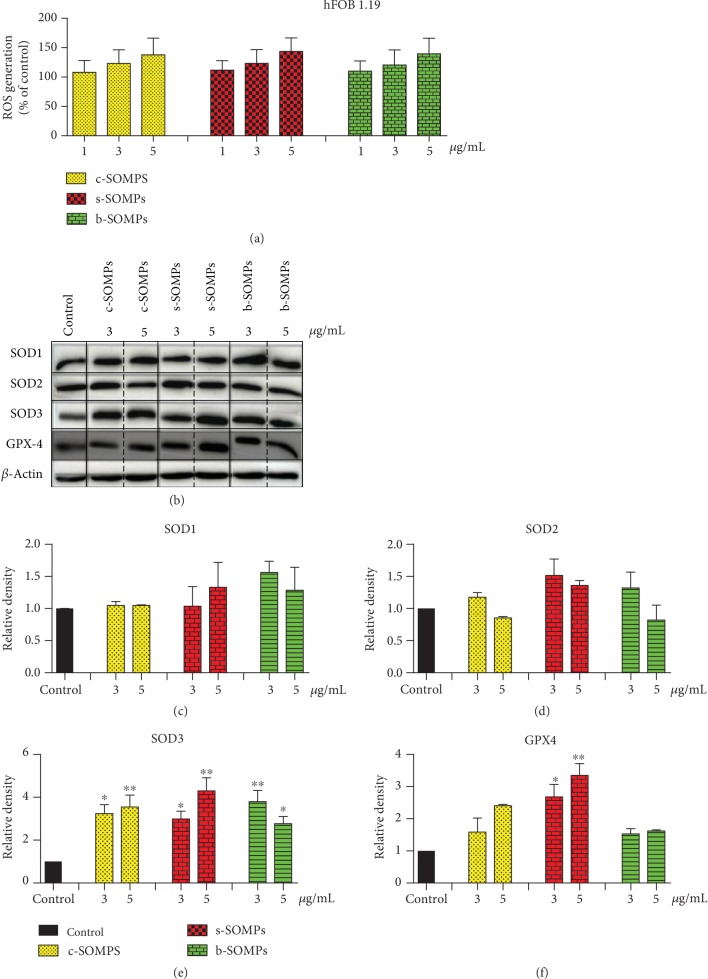
Impact of SOMPs on oxidative stress in hFOB1.19 cells. (a) ROS levels, (b) representative Western blots, (c) quantification of SOD1 levels, (d) quantification of SOD2 levels, (e) quantification of SOD3 levels, and (f) quantification of GPX4 levels in hFOB1.19 cells after incubation with c-SOMPs, s-SOMPs, or b-SOPMs for 24 h. Data are presented as mean ± SD. ^∗^*p* < 0.05, ^∗∗^*p* < 0.01, ^∗∗∗^*p* < 0.001.

Increased ROS production was seen in C2C12 and absent in hFOB1.19 cells (Figures 5(a) and 6(a)). Only c-SOMPs and s-SOMPs, both in 5 *μ*g/mL concentration, statistically significant increased level of ROS in C2C12 cells. We also examined levels of oxidative stress-related proteins. SOD1 levels in hFOB1.19 cells were not significantly changed (Figure 6(c)). SOD1 levels were only increased when C2C12 cells were treated with c-SOMPs (3 *μ*g/mL). Interestingly, the same shape of silver orthophosphate in higher concentration (5 *μ*g/mL) decreased levels of SOD1 (Figure 5(c)). Our SOMPs did not impact SOD2 levels (Figures 5(d) and 6(d)). All tested shapes (c-SOMPs, s-SOMPs, b-SOPMs) in all concentrations increased the levels of SOD3 in hFOB1.19 cells (Figure 6(e)). c-SOMPs (in 3 *μ*g/mL concentration) and b-SOMPs (in 5 *μ*g/mL concentration) increased levels of SOD3 in C2C12 cells (Figure 5(e)). In hFOB1.19 cells, GPX4 levels were increased after incubation with s-SOMPs (3 and 5 *μ*g/mL), whereas in C2C12 cells after treatment with 3 and 5 *μ*g/mL c-SOMPs (Figures 5(f) and 6(f)).

### 3.7. Impact of SOMPs on MMP1, MMP3, p16-ARC, and NF-*κ*B Levels

**Figure 7:**
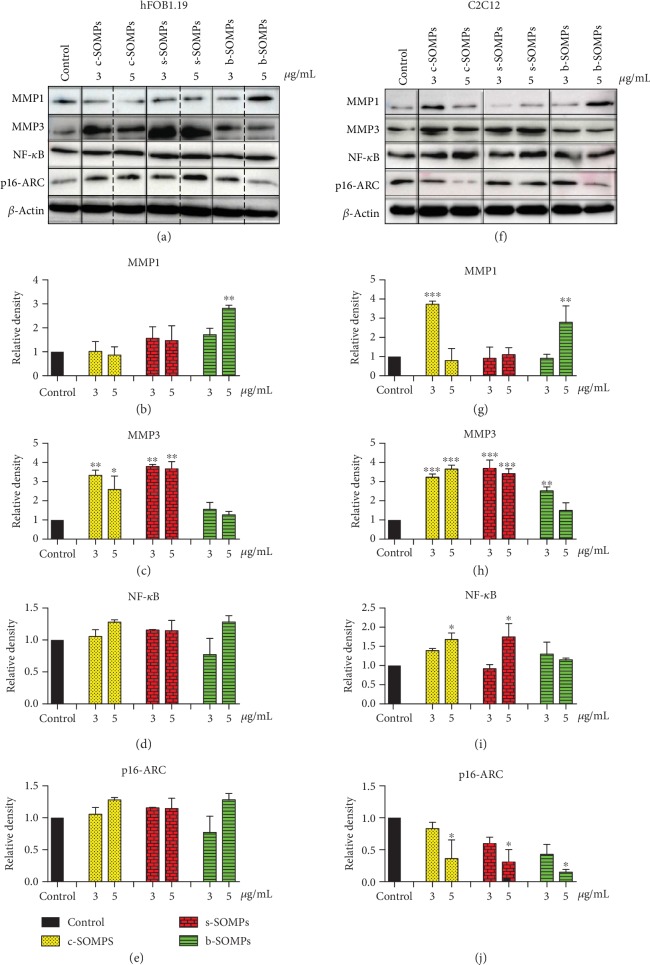
Impact of SOMPs on selected proteins levels. Representative Western blot analysis of (a) hFOB1.19 and (b) C2C12 cells after 24 h incubation with c-SOMPs, s-SOMPs, or b-SOPMs. Quantitative analysis of (b), (g) MMP1; (c), (h) MMP3; (d), (i) NF-*κ*B, and (e); and (j) p16-ARC levels on hFOB1.19 and C2C12 cell lines, respectively. Data are presented as mean ± SD. ^∗^*p* < 0.05, ^∗∗^*p* < 0.01, ^∗∗∗^*p* < 0.001.

Figure 7 presents the impact of SOMPs on MMP1, MMP3, p16-ARC, and NF-*κ*B levels. Our microparticles increased levels of MMP1 and MMP3 proteins. MMP1 levels were elevated when hFOB1.19 cells were treated with 5 *μ*g/mL of b-SOMPs and when C2C12 cells were incubated with 3 *μ*g/mL of c-SOMPs or 5 *μ*g/mL of b-SOMPs (Figures 7(b) and 7(g)). c-SOMPs (3 and 5 *μ*g/mL) and s-SOMPs (3 and 5 *μ*g/mL) increased levels of MMP3 in both cell lines (Figures 7(c) and 7(h)). Moreover, b-SOMPs (3 *μ*g/mL) increased levels MMP3 on C2C12 cells. NF-*κ*B levels were elevated in C2C12 cells were treated with 5 *μ*g/mL of c-SOMPs or s-SOMPs (Figures 7(d) and 7(i)). p16ARC levels were decreased in C2C12 cells after incubation with c-SOMPs, s-SOMPs, or b-SOMPs in 5 *μ*g/mL concentration (Figures 7(e) and 7(j)).

### 3.8. Analysis of Cell Cycle

**Figure 8:**
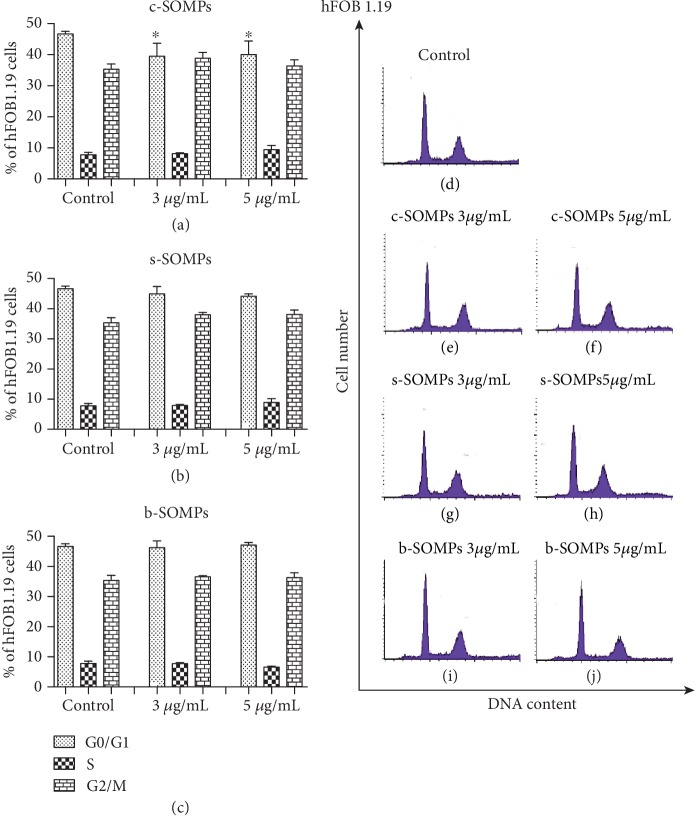
The cell cycle distribution for hFOB 1.19 cells. Percentage of cells in each cell cycle phase after treatment with (a) c-SOMPs, (b) s-SOMPs, and (c) b-SOMPs. Representative histograms (d) control, (e), (f) c-SOMPs; (g), (h) s-SOMPs; and (i), (j) b-SOMPs. Data are presented as mean ± SD. ^∗^ *p* < 0.05.

**Figure 9:**
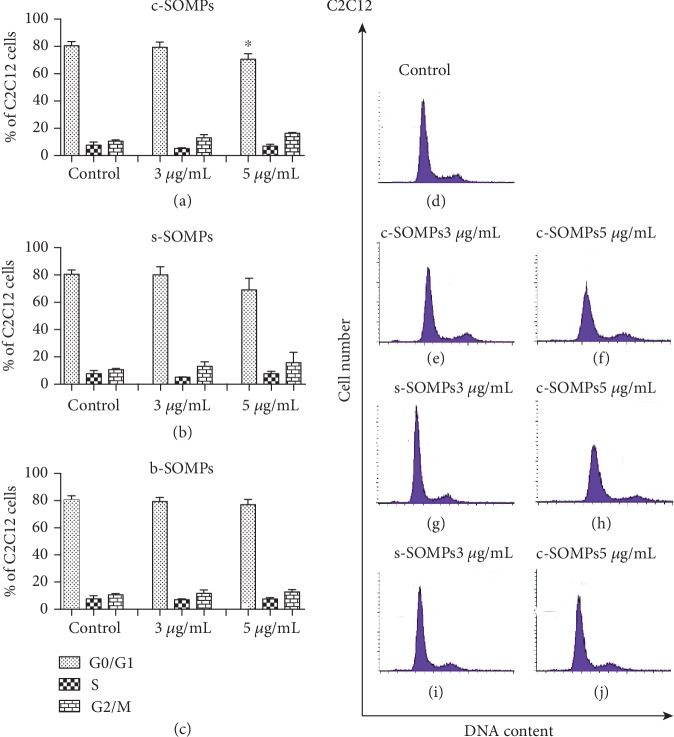
The cell cycle distribution for C2C12 cells. . Percentage of cells in each cell cycle phase after treatment with (a) c-SOMPs, (b) s-SOMPs, (c) b-SOMPs. Representative histograms (d) control, (e), (f) c-SOMPs; (g), (h) s-SOMPs; (i), (j) b-SOMPs. Data are presented as mean ± SD. ^∗^*p* < 0.05.

c-SOMPs statistically significantly decreased percentage of hFOB 1.19 cells in G0/G1 phase, in 3 and 5 *μ*g/mL concentrations (Figure 8). Moreover, c-SOMPs in a concentration of 5 *μ*g/mL statistically significantly decreased percentage of C2C12 cells in G0/G1 phase (Figure 9). Other changes in cell cycle distribution were not observed (Figures 8 and 9). s-SOMPs and b-SOMPs have no impact on the cell cycle distribution of hFOB1.19 and C2C12 cells.

### 3.9. TEM Analysis

**Figure 10:**
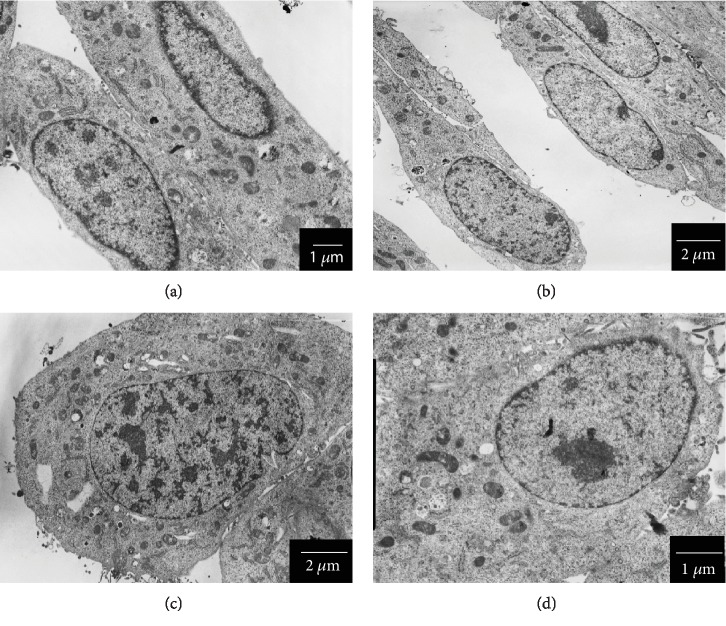
Morphology of C2C12 cells examined by TEM. (a) control cells, (b) cells treated with c-SOMPs (3 *μ*g/mL), (c) cells treated with s-SOMPs (3 *μ*g/mL), and (d) cells treated with b-SOMPs (3 *μ*g/mL). Scale bar is present on the bottom right side of each picture.

TEM analysis (Figure 10) has shown that c-SOMPs, s-SOMPs, and b-SOPMs in 3 *μ*g/mL concentration are not internalized by the C2C12 cells. Furthermore, we did not observe any ultrastructure changes within the cells.

## 4. Discussion

In the study, we synthesized and assessed SOMPs as a potential biomaterial. Antimicrobial properties and safety of potential application were taken into concern. We have shown that cytotoxicity and antimicrobial properties were shape- and concentration-dependent. Furthermore, SOMPs can be harmful to bacteria and fungi in concentrations safe for mammalian cell lines. It is the first study in which SOMPs or SONPs were examined in mammalian cells an *in vitro* model. Also, data about the cytotoxicity of other MPs are very limited.

### 4.1. Antimicrobial Properties

Antibacterial agents can be separated into two groups: semiconductors and metal-based ones. SOMPs belong to both, which greatly expand their antibacterial potential [35]. Thus, we hypothesized that SOMPs synthesized by our group will exhibit antimicrobial activity, which was confirmed experimentally. Moreover, shape-dependent antimicrobial properties of SOMPs were revealed. Among tested ones, c-SOMPs and s-SOPMPs were characterized by the highest activity. This should be emphasized that the tested SOMPs acted against both planktonic and biofilm forms of pathogens. Biofilm is a complex structure built from cells and extracellular matrix. It is known that pathogens in a biofilm are more resistant to treatment than planktonic forms [5]. Biofilm is poorly penetrated by antibiotics and immunological cells which makes its treatment a daunting challenge [2, 4, 5]. Furthermore, biofilm can easily be formed on foreign bodies that intruded into the human body, so it is clear that it is a major clinical problem [4]. Therefore, we decided to measure MBEC in addition to MIC. We focused on four pathogens: *S*. *aureus*, *MRSA*, *C*. *albicans*, and *A*. *niger*. Selected pathogens are well known as an etiological factor of bone- and/or implant-related infections [2, 3, 36, 37]. As a matter of fact, only a few studies have examined the antibacterial properties of SOMP, while antifungal and antibiofilm effects have not been previously reported.

Panthi et al. have shown that 200 nm SOMPs can be effective against *S*. *aureus*, *Escherichia coli*, *Klebsiella pneumoniae*, and *Pseudomonas aeruginosa* [38]. Also, Chudobova et al. have shown that 200-300 nm silver orthophosphate particles can be effective against *S*. *aureus* with the half-maximal inhibitory concentration equals 268.2 *μ*M [39]. While Liu et al. have reported the effectiveness of SOMPs against *E*. *coli* (DH-5*α*) [40]. However, the tested SOMPs decreased the viability of *E*. *coli* only at a concentration range of 10-100 *μ*g/mL, which means they are less effective than SOMPs described in this study. On the other hand, Yeo et al. have shown that c-SOMPs are more effective than rd-SOMPs against *E*. *coli* which is consistent with our data [41]. That phenomenon was explained by the fact that c-SOMPs are able to release more Ag^+^ ions than rd-SOMPs [41]. Furthermore, they found that SOMPs exhibit better antibacterial activity compared to similar structures made from Ag_2_O or CuO [41]. In fact, several mechanisms of the antimicrobial properties of SOMPs are described (Table 5). It should be highlighted that Ag_3_PO_4_ itself in a concentration of 5 *μ*g/mL can inhibit the growth of *S*. *aureus* as well [42].

### 4.2. Cytotoxicity Screening

In the study, we decided to use three cell lines as a bone model. Apart from human fetal osteoblasts (hFOB1.19) and mouse preosteoblast (MC3T3-E1), osteosarcoma cells were also used (Saos-2). Although derived from cancer often, Saos-2 cells are used as a bone cell model [43, 44]. Skin and muscle cells (HDF and C2C12 cells) were also used in cytotoxicity screening, as models of tissues which can potentially come in contact with SOMP-coated implant. We decide to use as many as 5 different cell lines and two different assays (MTT and BrdU), as it is proven to increase the quality and reliability of cytotoxicity screening [45, 46]. MTT assays estimate cell viability by measuring mitochondrial metabolism, whereas BrdU assays assess cell proliferation and DNA synthesis by determining 5-bromo-2′-deoxyuridine incorporation [45, 47]. Both MTT and BrdU have shown similar results. Generally, in the same conditions, cytotoxicity assessed by BrdU was higher than that assessed by MTT assay, which is consistent with literature data comparing those assays [48].

We clearly have shown that the cytotoxicity of our SOMPs was shape-dependent. c-SOMPs were the most cytotoxic ones, whereas t-SOMPs had the smallest effect on cell viability. Also, the response of different cell lines varies. SOMPs had the highest effect on the viability of C2C12 cells and the smallest on hFOB1.19 (based on IC_50_ comparison). Motskin et al. have examined the impact of 2-3 *μ*m hydroxyapatite MPs on human monocytes-macrophages (HMM), as they used MTT assay. They have shown concentration- and size-dependent cytotoxicity of MPs. The bigger the MPs were, the less cytotoxic they were [49]. He et al. have made a similar conclusion; however, they used spherical mesoporous silica MPs [50]. In our study, the biggest t-SOMPs were also the least cytotoxic. However, their nanoparticles have shown a significant decrease in cell viability in >250 *μ*g/mL concentration [49].

### 4.3. Oxidative Stress Induction

SOMPs are known to release free electrons, therefore, inducing ROS productions and oxidative stress [40]. ROS are the byproduct of metabolism and also can be used by cells as signalling molecules. However, the increased level of ROS can be lethal [51]. Excess of ROS can disturb cellular homeostasis and that condition is commonly called oxidative stress [51]. Several protein levels can be changed when oxidative stress occurs. NF-*κ*B (nuclear factor kappa-light-chance-enhancer of activated B cells) is a transcriptional factor involved in physiological regulations as well as in response to injury. Moreover, NF-*κ*B can be activated by ROS [52]. SOD1, SOD2, SOD3, and GPX4 are part of an antioxidative system of the cells [52]. Hence, we decided to examine the impact of selected SOMPs on ROS, levels, and expression on selected oxidative-stress response proteins: SOD1, SOD2, SOD3, GPX4, and NF-*κ*B. Our SOMPs increased ROS production. Also, we observed changes in SOD1, SOD3, GPX4, and NF-*κ*B. SOD2 levels were not affected. Mainly levels of mentioned proteins were elevated, with one exception. Interestingly, SOD1 levels in C2C12 could be either increased or decreased with regard to c-SOMPs concentration (more detailed description in sections 3.6 and 3.7 of the manuscript). We suggest that when oxidative stress is mild and not prolonged antioxidative protein levels will be increased (upregulation in order to fight danger). However, prolonged or intensive oxidative stress can impair the functioning of the cells, causing a decrease in protein levels. Our hypothesis is consistent with literature data [53]. Therefore, we suggest that SOMPs in some condition can trigger oxidative stress. A similar observation had been made for other MPs. Santos et al. have shown that different sizes of porous silica microparticles in >1 mg/mL concentration can trigger ROS production in human colon carcinoma (Caco-2) cells [54]. Also, AgNPs could increase ROS production in a shape-dependent manner [55]. The highest amount of ROS were produced by human fibroblast cells after treatment with 12.8 nm triangular AgNPs [55].

### 4.4. Impact at Proinflammatory Proteins

An implant as any foreign body may cause inflammation [56]. In order to examine if our SOMPs can trigger inflammation, we examined three proteins: NF-*κ*B, MMP-1, and MMP-3. NF-*κ*B was mentioned above as its levels can be changed in response to the excess of ROS [52]. However, this transcription factor has several roles and it is crucial for the inflammatory response [57]. MMP-1 and MMP-3 are collagen destruction enzymes which are elevated when inflammation occurs [58]. We have shown that some SOMPs increased levels of NF-*κ*B, MMP-1, and MMP-3. It may suggest that they act as proinflammatory agents. Similar to our findings, literature data suggest that Ag_3_PO_4_ in 50 *μ*g/mL concentration in human non-small-cell lung carcinoma cells (H1299) can increase levels of IL-8, which is a proinflammatory cytokine [42].

### 4.5. Impact on Cell Cycle

Cell division is crucial for proper wound healing, so possible antimicrobial agents to be used on implant should interfere with the cell cycle. In our study, only c-SOMPs decreased the percentage of cells in G0/G1 phase. Other MPs also can cause changes in the cell cycle. Chinde et al. have shown that tungsten oxide MPs can increase percentages of cells in G2/M phases in human lung carcinoma cells (A549) [59].

### 4.6. Internalization, Ultrastructure Changes, and Impact on the Cytoskeleton

We performed TEM analysis in order to assess whether SOMPs are internalized or caused any changes in cell ultrastructure. We saw deletion in p16-ARC levels; however, any visible changes in cells morphology were observed. p16-ARC is protein involved in actin polymerization, thus cytoskeleton formation. SOMPs were also not internalized by C2C12 cells. Motskin et al. have shown that HMM cells can internalize 2-3 *μ*m hydroxyapatite MPs. However, their study was performed on macrophages, which biological functions are based on ability to phagocytosis; so, they are more likely to uptake large particles [49]. They also used much greater concentration (125 *μ*g/mL) compared to our experiments. Similarly, He et al. have shown that mesoporous silica microparticles can be internalized into lysosomes. They conducted a study on mammary gland adenocarcinoma cells (MDA-MB-468). Again, they used a higher concentration than that used in our study [50].

### 4.7. Safety of Potential Applications

Our SOMPs have antimicrobial properties. Importantly, they can be selectively cytotoxic to bacteria and fungi and still be not harmful to mammalian cells. However, like any medication, they have also a side effect. In a higher concentration, they are cytotoxic to a mammalian cell. Also, they can induce inflammation and oxidative stress. Silver itself also can be noxious to mammalian cells. Unfortunately, there are no international standards regarding safe silver nano- or microparticle concentrations for humans. According to the U.S. Environmental Protection Agency, National Center for Environmental Assessments, an oral dose of 0.014 mg/kg/24 h of silver can be harmful and cause argyria [60]. However, due to much smaller doses and only local administration, it is highly unlikely that silver-coated implants can cause any adverse effect due to silver overdose. Moreover, nowadays, silver is commonly used in dressings with only one cause of argyria being reported (in an individual with 30% skin burnt) [61], which further support the safety of local application of silver. Commonly used antimicrobial agents also can decrease cell viability. For example, broad-spectrum antibiotic polymyxin B in 50 *μ*g/mL concentration reduces the viability of human erythroleukemia cells (K562) by one-fifth [62]. Wang et al. have shown that amphotericin B, colistin-M, and amikacin can decrease viability, measured by MTT assay, of pig corneal epithelial cells [63]. Duewelhenke et al. have shown that other clinically used drugs (cefazolin, ciprofloxacin, tetracycline, rifampicin, clindamycin, azithromycin, chloramphenicol, linezolid) can be cytotoxic to primary human osteoblasts (PHO), MG63 osteosarcoma (MG-63) and cervical cancer (HeLa) cells [64]. They observed decreased viability (MTT assay) and cell proliferation (BrdU assay) [64]. Their results are especially relevant for us because in the study they used a similar methodology and *in vitro* model; moreover, they also examined antibiotics used in treating bone infections.

## 5. Conclusion

We synthesized and characterized six shapes of silver orthophosphate microparticles (tetrapod, cubes, spheres, tetrahedrons, branched, and rhombic dodecahedrons). SOPMs had antimicrobial properties (both on planktonic and biofilm forms of pathogens), they were more efficient against fungi than bacteria. c-SOMPs and s-SOMPs had the best antimicrobial properties. Cytotoxicity of SOMPs was shape- and concentration-dependent. hFOB1.19 cells were the most resistant and C2C12 cells were the most susceptible to tested SOMPs. c-SOMPs were the most cytotoxic and t-SOMPs the least. Some of SOMPs can induce oxidative stress and increased levels of proinflammatory markers in the cells. SOMPs did not cause ultrastructure changes in C2C12 cells.

Based on good antimicrobial properties, mild cytotoxicity, no impact on cell cycle, and ultrastructure of the cells, we gather that spheres are the best shape of the silver orthophosphate microparticles for potential biomedical usage.

## Figures and Tables

**Table 1:** Minimal inhibitory concentrations of SOMPs against reference strains of microorganism.

	MIC (*μ*g/mL)			
	*Staphylococcus aureus*	*Staphylococcus aureus* (MRSA)	*Candida albicans*	*Aspergillus niger*
t-SOMPs	64	16	8	8
c-SOMPs	8	8	4	1
s-SOMPs	8	8	4	8
th-SOMPs	16	16	8	8
b-SOMPs	32	32	8	4
rd-SOMPs	64	64	16	8

**Table 2:** Minimal biofilm eradication concentrations of SOMPs against reference strains of microorganism.

	MBEC (*μ*g/mL)			
	*Staphylococcus aureus*	*Staphylococcus aureus* (MRSA)	*Candida albicans*	*Aspergillus niger*
t-SOMPs	128	32	8	8
c-SOMPs	32	16	8	2
s-SOMPs	64	16	16	8
th-SOMPs	128	16	16	16
b-SOMPs	256	32	64	16
rd-SOMPs	512	64	63	32

**Table 3:** IC_50_ values for different shapes of SOMPs (MTT assay). The values are approximated to decimal parts.

	IC_50_ (*μ*g/mL) (MTT assay)				
	hFOB1.19	MC3T3-E1	Saos-2	C2C12	HDF
t-SOMPs	>10	>10	>10	7.34	>10
c-SOMPs	5.97	4.94	4.79	3.73	4.93
s-SOMPs	>10	7.60	8.01	4.84	8.17
th-SOMPs	>10	6.62	5.19	4.50	5.00
b-SOMPs	>10	>10	8.34	9.08	8.36
rd-SOMPs	>10	>10	>10	>10	>10

**Table 4:** IC_50_ values for different shapes of SOMPs (BrdU assay). The values are approximated to decimal parts.

	IC_50_ (*μ*g/mL) (BrdU assay)				
	hFOB1.19	MC3T3-E1	Saos-2	C2C12	HDF
t-SOMPs	>10	>10	>10	7.12	>10
c-SOMPs	7.88	6.60	6.67	2.50	4.82
s-SOMPs	8.14	6.24	7.27	3.19	8.48
th-SOMPs	7.64	8.13	7.69	4.59	8.44
b-SOMPs	8.29	7.17	8.55	8.39	7.54
rd-SOMPs	>10	5.88	7.06	7.58	>10

**Table 5:** Possible mechanism underlying antibacterial properties of SOMPs.

Mechanism	Reference
Due to large surface and high surface energy, SOMPs can absorb bacteria	[38, 40]
SOMPs may release Ag^+^ ions which themselves are antibacterial agents	[38, 40, 41]
SOPMs under visible light can generate free electrons, therefore generating ROS which can lead to DNA damage	[38, 40]
PO_4_^3-^ ions can be released from SOMPs and interfere with ATP ← → ADP conversion, which will impair bacterial metabolism	[38, 40]

## Data Availability

The experimental data used to support the findings of this study are included within the article or are available from the corresponding author upon request.

## Abbreviations

SOMPs: Silver orthophosphate microparticles
t-SOMPs: Tetrapod silver orthophosphate microparticles
c-SOMPs: Cubical silver orthophosphate microparticles
s-SOMPs: Spherical silver orthophosphate microparticles
th-SOMPs: Tetrahedral silver orthophosphate microparticles
b-SOMPs: Branched silver orthophosphate microparticles
rd-SOMPs: Rhombic dodecahedral silver orthophosphate microparticles.

## Supplementary Materials

Supplementary Materials Supplementary Table 1: minimal inhibitory concentrations (*μ*g/mL) of conventional drugs against reference strains of microorganism.
