# Supplementary material for: Shape-Depended Biological Properties of Ag3PO4 Microparticles: Evaluation of Antimicrobial Properties and Cytotoxicity in In Vitro Model—Safety Assessment of Potential Clinical Usage
Source: Oxid Med Cell Longev. 2019 Nov 20;2019:6740325. doi: 10.1155/2019/6740325 (PMC6886340; doi:10.1155/2019/6740325)
Supplement: Supplementary Materials — Supplementary Table 1: minimal inhibitory concentrations (μg/mL) of conventional drugs against reference strains of microorganism. [file 6740325.f1.pdf]

Supplementary material

Shape-depended biological properties of Ag_3PO_4 microparticles. Evaluation of antimicrobial properties and cytotoxicity in *in vitro* model. Safety assessment of potential clinical usage

Karol P. Steckiewicz[1, 4], Julia Zwara[2], Maciej Jaskiewicz[3], Szymon Kowalski[1], Wojciech Kamysz[3], Adriana Zaleska-Medynska[2], Iwona Inkielewicz-Stepniak[1]

1- Department of Medical Chemistry, Medical University of Gdansk, Faculty of Medicine Gdansk, Poland
2-Department of Department of Environmental Technology, Faculty of Chemistry, University of Gdansk, Gdansk, Poland
3- Department of Inorganic Chemistry, Faculty of Pharmacy, Medical University of Gdansk, Gdansk, Poland
4- Department of Histology, Medical University of Gdansk, Faculty of Medicine Gdansk, Poland

correspondence should be address to Iwona Inkielewicz-Stepniak; iinkiel@gumed.edu.pl

Supplementary Table 1: Minimal Inhibitory Concentrations [µg/mL] of conventional drugs against reference strains of microorganism.

	S. aureus **ATCC 25923**	***S. aureus*** **ATCC 33591**		***C. albicans*** **ATCC 10231**	***A. niger*** **ATCC 16404**
Ampicillin	0.5	> 128	Fluconazole	2	8
Ciprofloxacin	1	0.25			
Linezolid	1	1	Nystatin	2	8
Vancomycin	1	1			
